# Post-extubation negative pressure pulmonary edema following acute upper airway obstruction in a young athlete: a case report

**DOI:** 10.1186/s13256-026-06565-z

**Published:** 2026-09-09

**Authors:** Mehdi Oudrhiri Safiani, Hamza Zarouali, Hamza Berrada, Omar Megane, Rim Essaoud, Othmane Sebbata, Bouchra Armel, Rachid El Moussaoui

**Affiliations:** 1 Department of Anesthesiology and Adult Intensive Care, Faculty of Medicine and Pharmacy of Rabat, Moulay Abdellah Hospital, Salé, Ibn Sina University Hospital Center (CHU Ibn Sina), Mohammed V University, Rabat, Morocco; 2 Department of Emergency, Intensive Care Medicine and Anesthesiology, Faculty of Medicine and Pharmacy of Rabat, Moulay Abdellah Hospital, Salé, Ibn Sina University Hospital Center (CHU Ibn Sina), Mohammed V University, Rabat, Morocco

**Keywords:** Pulmonary edema, Airway obstruction, Anesthesia recovery period, Ultrasonography, Noninvasive ventilation

## Abstract

**Background:**

Negative pressure pulmonary edema (NPPE) is a non-cardiogenic hemodynamic pulmonary edema resulting from significant inspiratory effort against obstructed airways. Although rare, it is a recognized cause of postoperative respiratory distress and can mimic differential diagnoses. Patients most predisposed to this condition are muscular or athletic individuals, due to their ability to generate significant negative intrathoracic pressure.

**Case presentation:**

An 18-year-old male patient underwent a laparoscopic appendectomy under general anesthesia with tracheal intubation. Two minutes after extubation, the patient developed acute respiratory distress with salmon-pink sputum. Lung auscultation revealed bilateral crackles, associated with desaturation and cyanosis, occurring in the context of acute obstruction of the upper airways during anesthetic awakening. A pleuro-pulmonary ultrasound performed according to the Bedside Lung Ultrasound in Emergency (BLUE) protocol showed a bilateral B-line pattern with preserved pleural sliding. Transthoracic echocardiography showed preserved left ventricular function with non-elevated filling pressures, allowing the rapid exclusion of a cardiogenic origin. Chest radiography demonstrated perihilar alveolar opacities consistent with pulmonary edema. A diagnosis of negative pressure pulmonary edema was thus made. Management consisted of a semi-sitting position at 30°, oxygen therapy, and non-invasive ventilation, without the use of diuretics. Clinical improvement was observed within the first 2 hours, with normalization of blood gases and radiological improvement within 24 hours.

**Conclusion:**

Negative pressure pulmonary edema (NPPE) should be considered a cause of post-extubation respiratory distress, particularly in young muscular patients. The integration of pleuro-pulmonary ultrasound and transthoracic echocardiography allows for rapid diagnostic guidance at the patient's bedside. Positive pressure ventilation remains the cornerstone of treatment.

## Introduction

Negative pressure pulmonary edema (NPPE) is a non-cardiogenic hemodynamic pulmonary edema resulting from significant inspiratory effort against an obstructed upper airway [1, 2]. This cause of postoperative respiratory distress most often affects young, muscular individuals due to their ability to generate significant negative inspiratory pressure [3].

This negative intrathoracic pressure increases venous return and transmural pulmonary capillary pressure, inducing a hydrostatic imbalance that leads to fluid leakage at the alveolar level [4].

There are two main types of NPPE [2, 5]. Type 1 occurs following acute upper airway obstruction, such as laryngospasm or endotracheal tube bite, and is most often seen in the peri- and postoperative periods. Type 2 is more frequently observed in the context of chronic upper airway obstruction. The reported incidence of type 1 NPPE is estimated to be between 0.05 and 0.1% of general anesthetics [2, 6]. This figure is likely an underestimate due to the rapid reversibility of this condition, which can easily go unnoticed.

The patients most at risk are young, healthy individuals without associated comorbidities who are capable of generating significant negative intrathoracic pressure during inspiratory effort with a closed glottis. The main triggering factor is postoperative laryngospasm [3].

The clinical presentation is often dramatic, combining acute postoperative respiratory distress with desaturation, significant dyspnea, signs of respiratory effort, and cyanosis, appearing a few minutes after the obstructive event [3].

Although the prognosis is generally favorable, with reversibility occurring within a few hours or days, this condition can cause severe hypoxia that may be life-threatening.

The main challenge for clinicians is to distinguish this condition from other causes of postoperative respiratory distress, including aspiration, cardiogenic pulmonary edema, acute respiratory distress syndrome (ARDS), residual neuromuscular blockade, and aspiration pneumonia, as management differs depending on the etiology. In this context, point-of-care ultrasound (POCUS) may represent a particularly valuable bedside diagnostic tool for rapidly orienting the etiology of postoperative respiratory distress in unstable patients.

We describe the case of an 18-year-old who developed postoperative respiratory failure immediately after extubation following a laparoscopic appendectomy. This case report highlights the importance of a systematic approach based on pleuro-pulmonary ultrasound using the Bedside Lung Ultrasound in Emergency (BLUE) protocol [7] to differentiate NPPE from other causes of postoperative respiratory distress. It also emphasizes the central role of positive pressure ventilation in the management of this condition.

## Case presentation

An 18-year-old male patient, weighing 75 kg and measuring 1.85 m in height (body mass index of 21.9 kg/m^2^), with no prior medical or surgical history, an athletic build, and a weightlifter, was admitted to the emergency department for acute right iliac fossa pain. Clinical, laboratory, and radiological investigations were consistent with acute appendicitis, indicating a laparoscopic appendectomy.

The pre-anesthetic examination revealed no abnormalities. Airway assessment showed no signs of intubation or difficult ventilation, with a Mallampati score of I, a thyromental distance greater than 6.5 cm, a mouth opening greater than 3.5 cm, a flexible neck, and no history of obstructive sleep apnea syndrome. The cardiovascular examination was unremarkable, with a normal electrocardiogram. The pleuro-pulmonary examination and chest X-ray were also normal. The patient was classified as American Society of Anesthesiologists (ASA) I E.

General anesthesia was chosen. Anesthetic induction was performed with intravenous propofol at a dose of 2.5 mg/kg, fentanyl at 3 µg/kg, and rocuronium at 0.6 mg/kg. Orotracheal intubation was straightforward, classified as grade I according to the Cormack–Lehane classification, and performed using a 7.5-mm cuffed endotracheal tube. Anesthesia was maintained with sevoflurane at a concentration of approximately one minimum alveolar concentration, with appropriate reinjections of the anesthetic drugs. Intraoperative monitoring included continuous electrocardiography, pulse oximetry, non-invasive blood pressure monitoring, capnography, and measurement of inspired and expired anesthetic gas concentrations. Quantitative neuromuscular blockade monitoring was not available in our operating room due to a lack of resources. The laparoscopic appendectomy was performed uneventfully and lasted approximately 55 minutes. The patient remained hemodynamically and respiratory stable throughout the procedure. Mechanical ventilation showed a peak inspiratory pressure of 30 cmH_2_O (related to the pneumoperitoneum) and a plateau pressure of 23 cmH_2_O. End-tidal CO_2_ remained normal throughout (32–36 mmHg), with no hypercapnia. Heart rate ranged between 60 and 80 bpm and blood pressure between 110/70 and 120/80 mmHg.

During emergence, while regaining consciousness and airway reflexes and still intubated, the patient repeatedly bit the tracheal tube against the oropharyngeal cannula, producing transient upper-airway obstruction. This was accompanied by a transient rise in peak airway pressure to 35–40 cmH_2_O, whereas end-tidal CO_2_ remained normal.

**Fig. 1:**
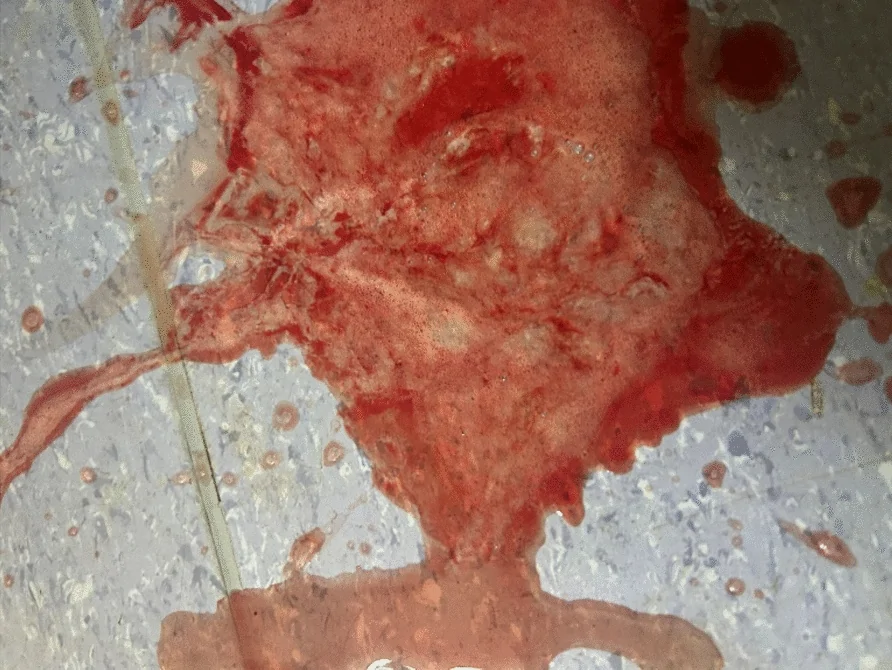
Pink frothy sputum observed immediately after extubation, suggestive of acute negative pressure pulmonary edema

At the end of surgery, after verifying the extubation criteria, including clinical reversal of neuromuscular blockade as demonstrated by a sustained head lift test lasting more than 5 seconds, preserved handgrip strength, spontaneous eye opening on command, and adequate tidal volumes, the patient was extubated fully conscious. The modified Aldrete score at the time of extubation was 10/10. Although no quantitative neuromuscular monitoring was available, all clinical criteria for extubation were met. Two minutes after extubation, the patient developed acute respiratory distress with dyspnea, salmon-pink sputum (Fig. 1), and bilateral crackles on lung auscultation. The frothy pink appearance of the sputum supported the hypothesis of acute pulmonary edema.

The patient presented with oxygen desaturation to 78% on room air, associated with tachypnea at 32 breaths per minute and perioral cyanosis. Hemodynamically, he presented with tachycardia at 122 beats per minute and a blood pressure of 140/80 mmHg. The patient remained neurologically stable with a Glasgow Coma Scale (GCS) score of 15.

Several diagnoses were considered, primarily negative pressure pulmonary edema (NPPE) given the sequence and chronology of events, the patient's characteristics (young, athletic, and muscular), and the onset of symptoms in the context of upper airway obstruction secondary to endotracheal tube bite. The second diagnosis considered was pulmonary aspiration, particularly in the context of emergency surgery with the risk of a full stomach. Other differential diagnoses were also discussed, including cardiogenic pulmonary edema, pneumothorax following positive pressure mechanical ventilation, massive atelectasis, acute respiratory distress syndrome (ARDS) due to severe hypoxemia, and residual neuromuscular blockade.

The management consisted of placing the patient in a semi-sitting position with their head tilted at 30°, combined with high-concentration oxygen therapy. Given the persistent respiratory distress and hypoxemia, non-invasive ventilation was initiated in pressure support mode with a pressure of 12 cmH_2_O, a positive end-expiratory pressure (PEEP) of 8 cmH_2_O, and a fraction of inspired oxygen (FiO2) of 0.6. The patient received continuous monitoring, including pulse oximetry, non-invasive blood pressure monitoring, continuous electrocardiography, and respiratory rate monitoring. Reintubation was not required.

**Fig. 2:**
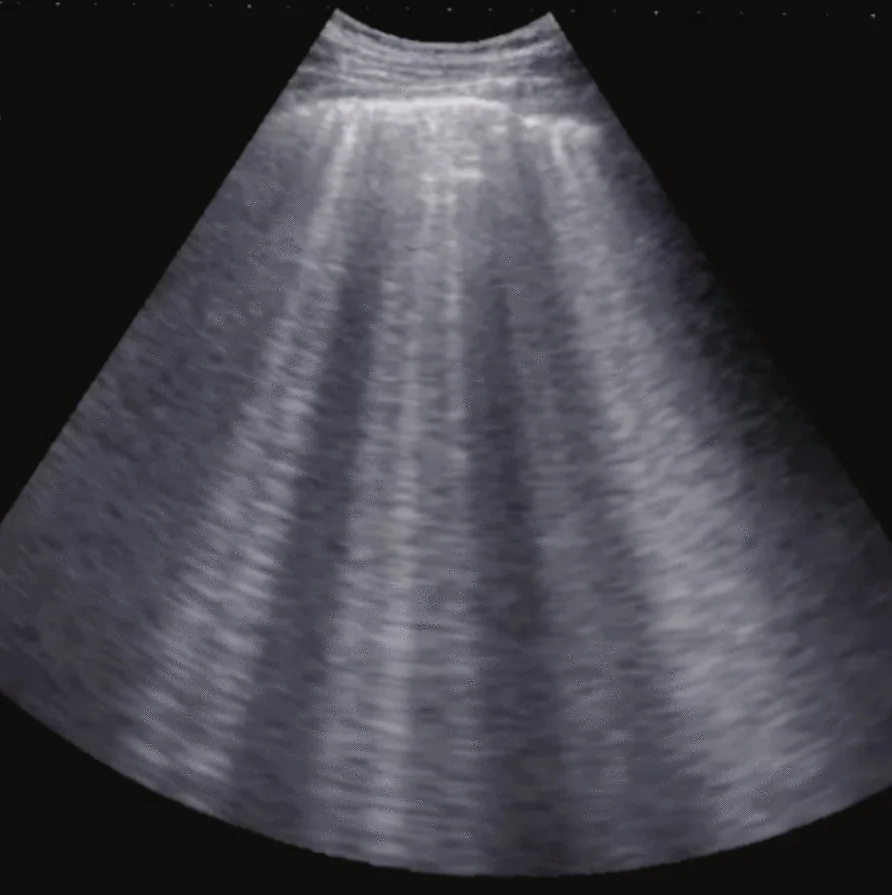
Lung ultrasound showing multiple confluent bilateral B-lines consistent with alveolo-interstitial syndrome in negative pressure pulmonary edema

Regarding the etiological investigation, arterial blood gas analysis performed under non-invasive ventilation (NIV) with n FiO2 of 0.6 revealed hypoxemia (58 mmHg) with a PaO2/FiO2 ratio of 97, associated with hypocapnia (33 mmHg) and a pH of 7.38. A pleuro-pulmonary ultrasound was performed within minutes of the onset of respiratory distress, in accordance with the BLUE protocol recommendations. The six anterior, superior lateral, and inferior lateral lung regions of each hemithorax were examined. The examination revealed diffuse bilateral lung involvement with bilateral B-lines throughout the lung fields examined. Pleural sliding was preserved with a regular and unfragmented pleural line. No signs of consolidation, subpleural irregularities, and no lung point were observed. These findings supported a diagnosis of alveolar-interstitial syndrome of hydrostatic origin and ruled out pneumothorax and massive atelectasis (Fig. 2).

**Fig. 3:**
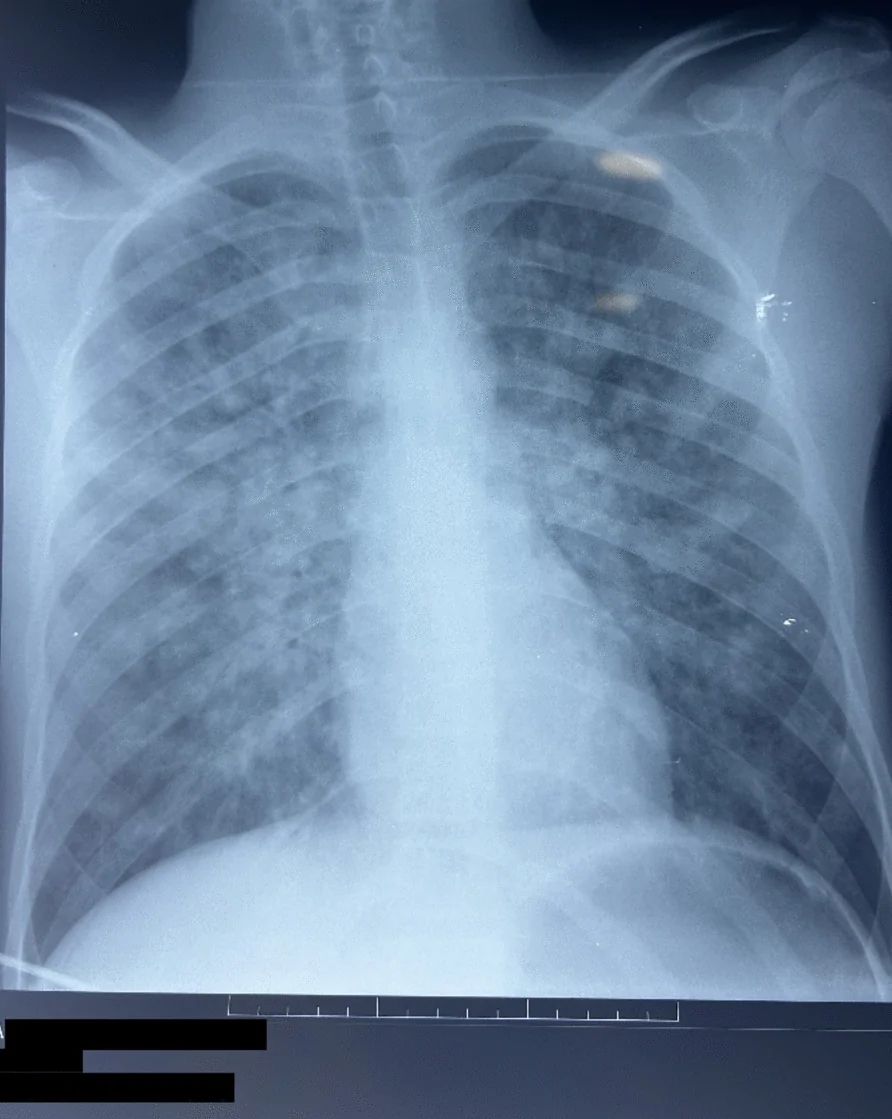
Chest X-ray demonstrating bilateral diffuse alveolo-interstitial infiltrates consistent with negative pressure pulmonary edema

Transthoracic echocardiography was performed to complement the pleuro-pulmonary echocardiographic evaluation. It showed preserved left ventricular function with an estimated left ventricular ejection fraction of 65%, without segmental or global wall motion abnormalities of the left ventricle. The right and left atria were not dilated. Filling pressures were not elevated, with an E/A ratio in the gray zone at 1.4 and an E/E' ratio of six. No significant valvular abnormalities were found. There was no tricuspid regurgitation or dilation of the inferior vena cava. All of these findings argued against a cardiogenic origin of the acute pulmonary edema. The chest X-ray revealed bilateral perihilar opacities with a normal cardiothoracic ratio, no pleural effusion, and no Kerley B-lines. No radiological signs suggestive of pulmonary aspiration were found (Fig. 3).

Therefore, after further investigation, we concluded that we had a diagnosis of negative pressure pulmonary edema syndrome (NPPE), based on several factors: the patient's profile: a young, athletic adult with well-developed musculature capable of generating significant negative inspiratory pressure; the circumstances of the onset, including a triggering factor of the endotracheal tube bite during the emergence phase of anesthesia; and the clinical course, with rapid onset 2 minutes after extubation and rapid resolution following the initiation of non-invasive positive pressure ventilation. The pleuro-pulmonary ultrasound findings were consistent with a bilateral B-shaped pattern with preserved pleural sliding and a regular pleural line, consistent with hydrostatic alveolar-interstitial edema. The echocardiogram was normal, ruling out a cardiogenic origin of the edema. The chest X-ray showed perihilar alveolar opacities with a normal cardiothoracic ratio, consistent with non-cardiogenic pulmonary edema.

Differential diagnoses were successively ruled out. Aspiration was considered unlikely despite the emergency setting and the risk of a full stomach, given the absence of any aspiration episodes during induction, intubation, or surgery, the presence of frothy sputum rather than true gastric contents during expectoration, and the chest X-ray showing no focal dependent involvement but rather perihilar opacities. Pneumothorax was ruled out due to the presence of preserved bilateral pleural sliding and a chest X-ray showing no pleural effusion. A cardiogenic origin of the pulmonary edema was ruled out based on an echocardiogram showing preserved systolic function with non-elevated filling pressures. Acute respiratory distress syndrome was ruled out due to the rapid resolution of respiratory distress within a single day, as well as the homogeneous distribution of B-lines on pleuro-pulmonary echography, unlike the typically heterogeneous ARDS pattern. Atelectasis was ruled out based on concordant findings from chest X-ray and pleuro-pulmonary ultrasound. Residual neuromuscular blockade could not be definitively ruled out due to the lack of neuromuscular monitoring. Nevertheless, clinical criteria for extubation were present, and the observed respiratory distress was inconsistent with simple residual neuromuscular blockade, which is usually responsible for impaired ventilation without associated pulmonary imaging.

Regarding the patient's condition, clinical improvement was rapid. Respiratory distress resolved within 12 hours with non-invasive ventilation, subsequently allowing for a gradual transition to simple oxygen therapy via face mask. The radiological evolution, with the disappearance of pulmonary edema within 24 hours, also supported this rapid recovery, as did the normalization of blood gases at 24 hours, with a PaO2 of 92 mmHg on room air and a PaCO2 of 40 mmHg.

The patient was transferred to the surgical ward after 24 hours and discharged on the fourth postoperative day.

The 1-month follow-up was normal, with the patient presenting no symptoms or respiratory sequelae (Table 1).

**Table 1:** Timeline of the patient's clinical course

Time	Event
T0	Extubation after recovery from general anesthesia
T + 2 minutes	Onset of dyspnea, pink frothy sputum, bilateral crackles, and severe oxygen desaturation (SpO_2_ 78%)
T + 3 minutes	30° head-up positioning and high-flow oxygen via non-rebreather mask (15 L/minute) initiated
T + 5 minutes	Bedside lung ultrasound and transthoracic echocardiography performed
T + 10 minutes	Non-invasive ventilation initiated (IPAP 12 cmH_2_O, PEEP 8 cmH_2_O, FiO_2_ 0.6)
T + 30 minutes	Transfer from the operating room to the intensive care unite
T + 2 hours	Marked clinical improvement with resolution of respiratory distress; weaning from NIV to oxygen mask
T + 24 hours	Normalization of arterial blood gases and near-complete radiological clearance
Day 2	Transfer from intensive care unit to the surgical ward
Day 4	Hospital discharge
1 month	Outpatient follow-up: no residual respiratory symptoms

cmH₂O centimeters of water, FiO₂ fraction of inspired oxygen, IPAP inspiratory positive airway pressure, L/min liters per minute, NIV non-invasive ventilation, PEEP positive end-expiratory pressure, SpO₂ peripheral oxygen saturation.

## Discussion

### Pathophysiology

Negative pressure pulmonary edema (NPPE) is a non-cardiogenic hemodynamic pulmonary edema resulting from a hydrostatic pressure imbalance leading to fluid leakage into the alveoli [1, 2]. This occurs when the patient is able to generate significant negative inspiratory pressures against obstructed or closed airways [3].

Indeed, intrathoracic pressures can reach very high negative values, such as − 50 or even − 140 cmH_2_O, much higher than the pressures reached during normal inspiration, which are typically − 2 to − 5 cmH_2_O [3].

This marked decrease in intrathoracic pressure has hemodynamic consequences. First, it induces an increase in venous return, increases transmural pulmonary pressure, increases left ventricular afterload, and triggers a sympathetic response with a redistribution of central blood volume toward the pulmonary circulation. All of this is consistent with an increase in pulmonary capillary hydrostatic pressure, which exceeds the limits of the alveolar–capillary barrier and thus induces fluid extravasation into the interstitium and alveoli [4]. Therefore, NPPE has often been described as the consequence of a purely hydrostatic mechanism, resulting primarily from hydrostatic pressure changes with preservation of capillary permeability.

However, studies have shown that stress failure of the alveolar–capillary barrier also contributes in the most severe cases, with increased barrier permeability through the disruption of both endothelial and epithelial cells [8]. This new understanding of NPPE may explain the occurrence of hemorrhagic pulmonary edema [9].

Clinically, this hydrostatic (in the most severe cases, hemorrhagic) nature of the alveolar fluid is reflected by the characteristic frothy, salmon-pink sputum observed in our patient (Fig. 1). Although highly suggestive, this sign is not specific to NPPE, as it may also occur in cardiogenic pulmonary edema.

However, it is still important to distinguish this mechanism from cardiogenic edema, where fluid extravasation results from elevated left ventricular filling pressures, which themselves lead to an increase in pulmonary capillary pressure. In NPPE, left ventricular function is preserved, and the edema resolves rapidly once the source of the obstruction is eliminated and positive pressure ventilation is initiated. Similarly, it must also be distinguished from ARDS, where there is a disruption of the alveolar–capillary barrier through a purely inflammatory mechanism.

Patients at highest risk of NPPE are young, muscular, and athletic, as this type of patient is capable of generating significant negative intrathoracic pressures against a closed glottis [3]. Other predisposing factors include obesity, obstructive sleep apnea syndrome, upper airway abnormalities, and any situation that promotes acute airway obstruction during the emergence phase of anesthesia. In our observation, young age, athletic build, and the occurrence of transient upper airway obstruction were the main predisposing factors [5].

### Literature review

The reported incidence of NPPE is around 0.05–0.1% of general anesthesia procedures (2.6). This figure is considered likely to be an underestimate due to unrecognized postoperative presentations.

In the case series and literature review conducted by Luo et al. [10], which analyzed cases of perioperative NPPE, it was found that the post-extubation phase accounts for the majority of NPPE cases. Furthermore, Luo et al. also highlighted a male predominance, younger age, and laryngospasm and endotracheal tube bite as the most frequent triggering factors [10].

The onset of NPPE is generally rapid, with most cases presenting with respiratory symptoms within minutes, or at most within an hour, following the obstructive event. The prognosis for this condition remains favorable when diagnosis is made early and treatment is appropriate and prompt [3].

Symptoms typically resolve within 12–48 hours, and the mortality rate remains relatively low. However, delayed diagnosis, inappropriate management with fluid resuscitation protocols, and the failure to initiate positive pressure ventilation can lead to profound and prolonged hypoxemia, an increased risk of superimposed infection, and progression to acute respiratory failure, which can be life-threatening [2, 10, 11].

In young adults, when NPPE is recognized as the cause of acute respiratory failure, the delay in patient management is considered the primary factor contributing to an unfavorable outcome [10].

Therefore, various reports concerning post-extubation pulmonary edema emphasize the role of point-of-care ultrasound (POCUS) at the bedside, allowing the identification of patterns characteristic of NPPE, namely the presence of homogeneous bilateral alveolar-interstitial syndrome patterns [7]. Cardiac ultrasound then allows for the exclusion of cardiac involvement, thus confirming a diagnosis of NPPE.

This use of both pulmonary and cardiac ultrasound in the post-extubation context for acute respiratory failure is recommended, especially when transporting the patient for a computed tomography (CT) scan or X-ray is considered impractical and potentially unsafe.

### Specific contribution of the present case

This case highlights two main points. First, it illustrates the value of combining pleuro-pulmonary ultrasound and transthoracic echocardiography to rapidly guide the diagnosis of postoperative respiratory distress and exclude the main differential diagnoses. Second, it shows that early management based on positive pressure ventilation may lead to a favorable outcome without reintubation or the systematic diuretic administration [12].

### Practical messages

Several practical points emerge from this case:

- NPPE should be systematically considered in any case of acute respiratory distress occurring after upper airway obstruction in the perioperative period, particularly in young, muscular, or athletic individuals.
- The combination of pleuro-pulmonary ultrasound and transthoracic echocardiography constitutes a first-line diagnostic tool allowing for rapid bedside triage and exclusion of the main differential diagnoses.
- Positive pressure ventilation represents the cornerstone of the treatment. Reintubation is only necessary in cases of refractory hypoxemia or clinical deterioration.
- Quantitative neuromuscular monitoring should be used when available to minimize the risk of residual neuromuscular blockade and improve extubation safety.

### Limitations

This case report presents several limitations.

Firstly, the diagnosis of NPPE is a presumptive diagnosis made after ruling out other possible diagnoses. Indeed, intrathoracic and transpulmonary pressures could not be measured in the emergency setting.

Secondly, lung ultrasound and transthoracic echocardiography are operator-dependent. Hence, proper interpretation of lung ultrasound and echocardiography requires an anesthesiologist/intensivist trained in POCUS.

Thirdly, the absence of quantitative neuromuscular monitoring did not allow formal exclusion of residual neuromuscular blockade. However, clinical extubation criteria were fulfilled, and the clinical presentation associated with the imaging findings made this hypothesis unlikely.

Finally, this is a unique observation whose conclusions cannot be generalized and must be interpreted in light of existing literature.

## Conclusion

In conclusion, negative pressure pulmonary edema is a rare but potentially serious complication of acute upper airway obstruction in the postoperative period. Its diagnosis is based on a combination of clinical and paraclinical evidence, with pleuro-pulmonary ultrasound combined with transthoracic echocardiography providing valuable assistance for rapid diagnostic guidance at the patient's bedside. Early recognition and rapid initiation of positive pressure ventilation generally allow for a favorable outcome.

## Data Availability

No datasets were generated or analyzed during the current study.

## Abbreviations

ARDS: Acute respiratory distress syndrome
ASA: American Society of Anesthesiologists
BLUE: Bedside Lung Ultrasound in Emergency
E/A: Ratio of early to late mitral inflow velocity
E/e′: Ratio of early mitral inflow velocity to early diastolic mitral annular velocity
FiO_2_: Fraction of inspired oxygen
GCS: Glasgow Coma Scale
IPAP: Inspiratory positive airway pressure
NIV: Non-invasive ventilation
NPPE: Negative pressure pulmonary edema
PaCO_2_: Partial pressure of carbon dioxide in arterial blood
PaO_2_: Partial pressure of oxygen in arterial blood
PEEP: Positive end-expiratory pressure
POCUS: Point-of-care ultrasound
SpO_2_: Peripheral oxygen saturation
